# MHD surrogate model for convection in electromagnetically levitated molten metal droplets processed using the ISS-EML facility

**DOI:** 10.1038/s41526-020-0099-7

**Published:** 2020-03-16

**Authors:** Evan B. Baker, Jannatun Nawer, Xiao Xiao, Douglas M. Matson

**Affiliations:** 1 Department of Mechanical Engineering, Tufts University, Medford, MA 02155 USA; 2 Institut für Materialphysik im Weltraum, German Aerospace Center (DLR), Linder Höhe, D-51147 Köln, Germany

**Keywords:** Aerospace engineering, Techniques and instrumentation

## Abstract

Electromagnetic levitation experiments in space are an essential tool for thermophysical property measurement and solidification studies. In light of the need for material properties as inputs to industrial process modeling, investigators need new tools for efficient experiment planning. MHD surrogate modeling is a parametric method for prediction of flow conditions during processing using the ISS-EML facility. Flow conditions in model Au, Zr, and Ti_39.5_Zr_39.5_Ni_21_ samples are predicted using the surrogate model. For Au, flow is shown be turbulent in nearly all experimental conditions, making property measurement difficult. For Zr, the flow is turbulent with the heater on and laminar with the heater off, allowing for property measurement during free-cooling experiments only. For TiZrNi, the flow is laminar under all experimental conditions, indicating that TiZrNi is an excellent candidate for EML experiments. This surrogate modeling approach can be easily applied to other materials of interest, enabling investigators to choose materials that will perform well in levitation and to tailor experiment parameters to achieve desirable flow conditions.

## Introduction

Numerical simulation of complex metal processing is important for manufacturing in industries such as aerospace, marine, nuclear reactors, and industrial gas turbine production. Precise knowledge of thermophysical properties improves modeling by increasing the accuracy of the input parameters. Electromagnetic levitation (EML) is a containerless processing method which enables high-temperature bulk materials processing in an undercooled state for an extended period. In microgravity, gravity-induced sample deformation is virtually eliminated, and the use of a spherical sample improves measurement fidelity. Space electromagnetic levitation facility (ISS-EML) samples are levitated using a SUPOS coil (a German acronym for superposition of frequencies) such that eddy currents are induced in the sample providing heating and positioning functions at different frequencies. When the magnetic field is imposed on the sample, Lorentz forces induce fluid flow in the levitated sample. Conducting experiments in microgravity environment reduces the buoyancy and positioning-driven flow in the levitated molten metal as compared to ground-based experiments. As a result, the sample stirring is significantly lower than in ground-based testing.

This is important because the measured thermophysical properties and phase selection of the samples are greatly influenced by convection^1^. Stirring deleteriously affects the accuracy of viscosity, surface tension, and thermal conductivity measurements. Therefore, a better understanding of convection leads to better accuracy and precision in thermophysical property analysis. EML samples are opaque and almost featureless. They are highly reactive at high temperature and the large alternating magnetic field makes it very difficult to directly measure velocity within a liquid sample^2^. The momentum transferred by eddies in the turbulent regime yields higher damping, which gives an erroneous measurement of viscosity^3,4^. Thus, it is critical to conduct the experiments in such conditions that the melt convection remains in the laminar regime. Many researchers have tried to approach this problem with analytical and numerical methods^5–7^. Hyers^8^ evaluated Marangoni convection and natural convection using magnetohydrodynamic (MHD) modeling which involves both computational fluid dynamics (CFD) and electromagnetic simulation tested by TEMPUS EML. Lee et al.^9^ developed a numerical model of convection in EML droplets and validated the simulated results with experimental data. However, it is very difficult to find quantitative validation of convection velocity beyond these publications.

Xiao et. al.^10^ developed a surrogate model to correlate convection in an electromagnetically levitated molten droplet by performing data sampling from the MHD simulation results. This model addresses both electromagnetic and fluid mechanics problems involved in the sample. EML samples can have a wide range of fluid velocity under different settings resulting in anywhere between laminar and turbulent regime^11^. Xiao^12^ also performed MHD simulation using a laminar model and an RNG k-ε turbulence model to predict flow parameters in both laminar and turbulent regimes. This generalized model can be applied to a wide range of material process testing performed in the ISS-EML facility. Important parameters such as the fluid velocity and shear rate can easily be calculated by using the results of the MHD surrogate model.

In this study, we show the ease of applicability of this model to develop test conditions for future experiments. We have applied this model for two pure samples, Au and Zr, and one glass-forming alloy, Ti_39.5_Zr_39.5_Ni_21_. These three samples were chosen based on the availability of their thermophysical property data from both experimental and literature values^13–16^. These general models provide coefficients needed to measure fluid flow for various heating and positioning control voltage settings which can be used as an important planning tool for researchers. Our main approach is to evaluate thermophysical properties for a given temperature and voltage setting to predict flow velocity, shear rate, and Reynolds number.

## Results

### Gold (Au)

**Fig. 1:**
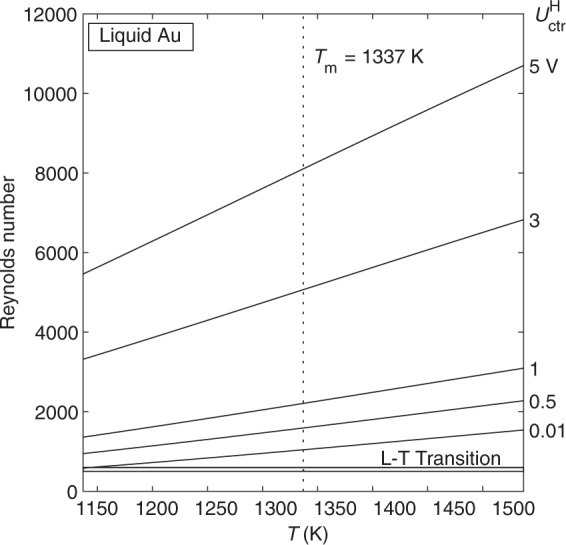
Reynolds number vs. temperature for flow in liquid Au as a function of heater control voltage, as predicted by the laminar model. Flow is turbulent (Re > 600) for every combination of temperature and voltage shown.

We first apply the surrogate model to analyze flow in liquid Au samples in EML. The Reynolds number is calculated as a function of temperature and heater control voltage (Fig. 1). As expected, the Reynolds number increases as temperature and heater voltage increase. At typical heater voltage levels, the Reynolds number greatly exceeds the laminar-turbulent transition. Even at very low heater voltage levels and deep undercooling, the Reynolds number is greater than 500. This indicates that flow in Au samples will always be turbulent when the heater is on.

**Fig. 2:**
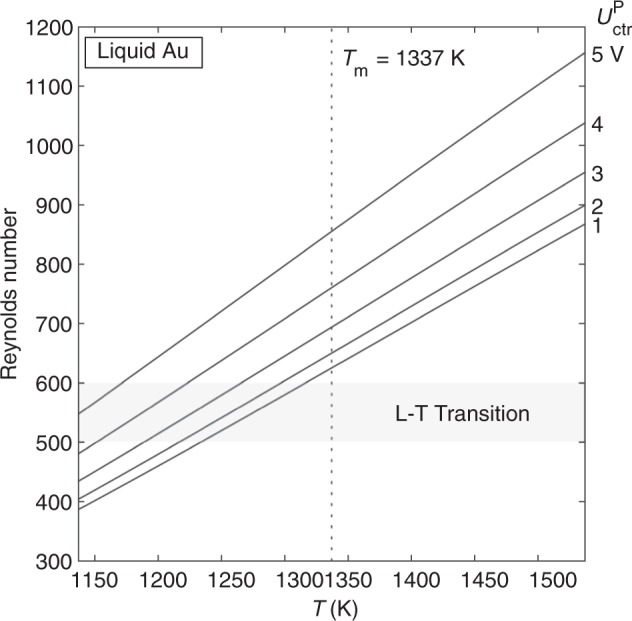
Reynolds number vs. temperature for flow in liquid Au as a function of positioner control voltage, as predicted by the laminar model. Flow is laminar when heater is off, positioner voltage is low, and undercooling is greater than 100 K.

Considering the preference for laminar flow, we can also look at the Reynolds number when the heater is turned off, and the positioner control is the dominant factor (Fig. 2). Flow in Au is still predicted to be turbulent under many common experiment conditions, even with the heater off. Laminar flow is only possible with low positioner voltage levels and undercooling greater than 100 K. Thus, experiments that require laminar flow will only be possible under carefully controlled conditions.

**Fig. 3:**
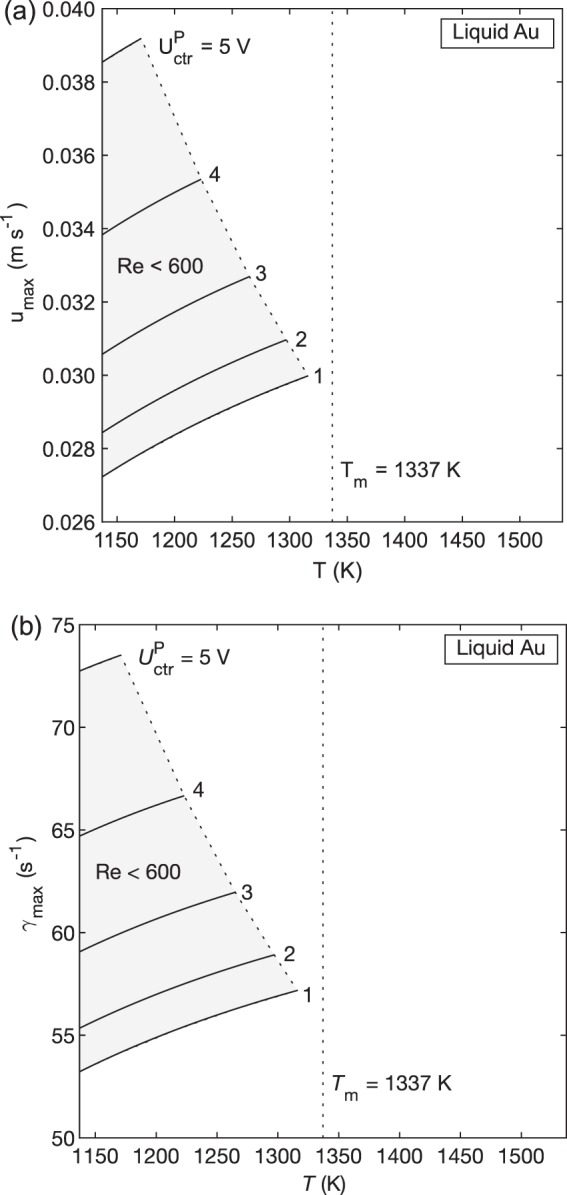
Convection in Au. Maximum velocity (**a**) and maximum shear rate (**b**) vs. temperature as a function of positioner control voltage. This shows the magnitude of stirring expected in Au samples as a function of temperature and positioner voltage. Curves terminate where the model becomes invalid due to the onset of turbulent flow, and the shaded area represents the valid region.

Maximum flow velocity (*u*_max_) and maximum shear rate ($\dot \gamma _{{\mathrm{max}}}$) are also calculated as a function of temperature and positioner control voltage (Fig. 3). For a given temperature and positioner voltage, we can therefore predict the amount of convection that will be present in a given sample during an experiment.

**Fig. 4:**
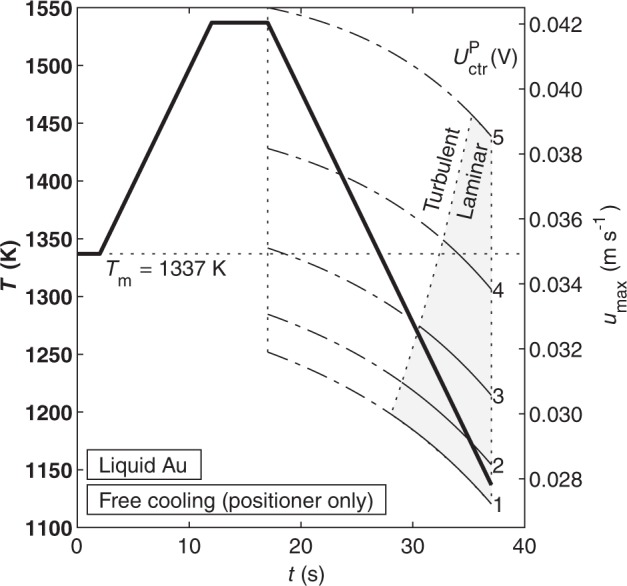
An idealized cycle in which a gold sample is melted, heated to T_m_ + 200, and then cooled with the heater off. Maximum flow velocity is plotted as a function of temperature and positioner control voltage. The shaded area represents the valid region where Re < 600.

To illustrate how the preceding results can aid in experiment design, we consider an idealized EML experiment and predict the flow conditions using the surrogate model (Fig. 4). As shown above, the flow in Au can only be laminar when the heater is off. Thus, we present an experiment in which the sample is melted, ramped to a 200 K superheat, and then allowed to cool freely with the heater off. If the positioner voltage can be kept as low as 1 V, the flow enters the L-T transition region at 1316 K (21 K undercooling) and becomes fully laminar at 1233 K (104 K undercooling). *u*_max_ = 0.0289 m s^−1^ and $\dot \gamma _{{\mathrm{max}}}$ = 55.6 s^−1^ at the point where the flow becomes fully laminar. If the positioner voltage requirements are 5 V, the flow does not become transitional until 1171 K (166 K undercooling) and fully laminar flow is likely not achievable. Because the Au sample must be allowed to cool freely in these experiments, there may only be a short time window in which laminar flow is present and the measured thermophysical property data is valid.

Overall, model results indicate that Au will be a difficult sample to work with in EML experiments. This is because laminar flow can only be achieved in a very restrictive set of conditions, including heater off, low positioner voltage, and undercooled samples. Even if the necessary conditions are achieved, they may be too limiting to satisfy the science objectives of the experiment.

### Zirconium (Zr)

**Fig. 5:**
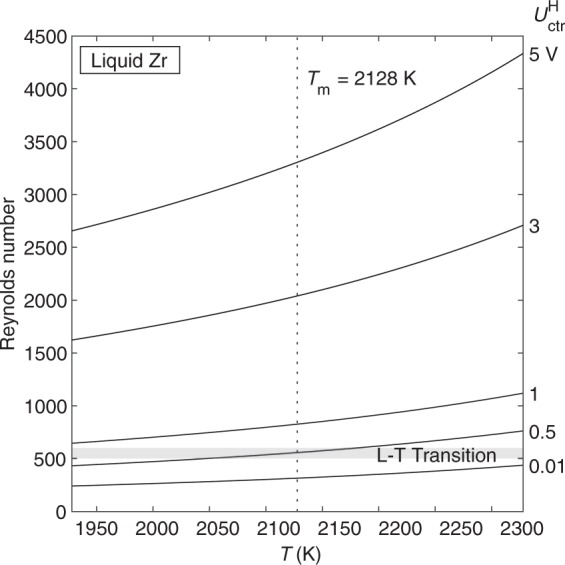
Reynolds number vs. temperature for flow in liquid Zr as a function of heater control voltage, as predicted by the laminar model. Laminar flow is accessible for $U_{\rm{ctr}}^{\rm{H}}$  = 0.01 V for the entire temperature range and for $U_{\rm{ctr}}^{\rm{H}}$ = 0.5 V at undercooling greater than 80 K.

Next, we consider flow in Zr. The surrogate model is again used to calculate the Reynolds number as a function of temperature and heater control voltage (Fig. 5). Re for Zr is considerably lower than that for Au. In contrast to Au, the model shows that laminar flow is accessible in Zr with the heater on, although $U_{{\mathrm{ctr}}}^{\mathrm{H}}$ must be kept very low.

**Fig. 6:**
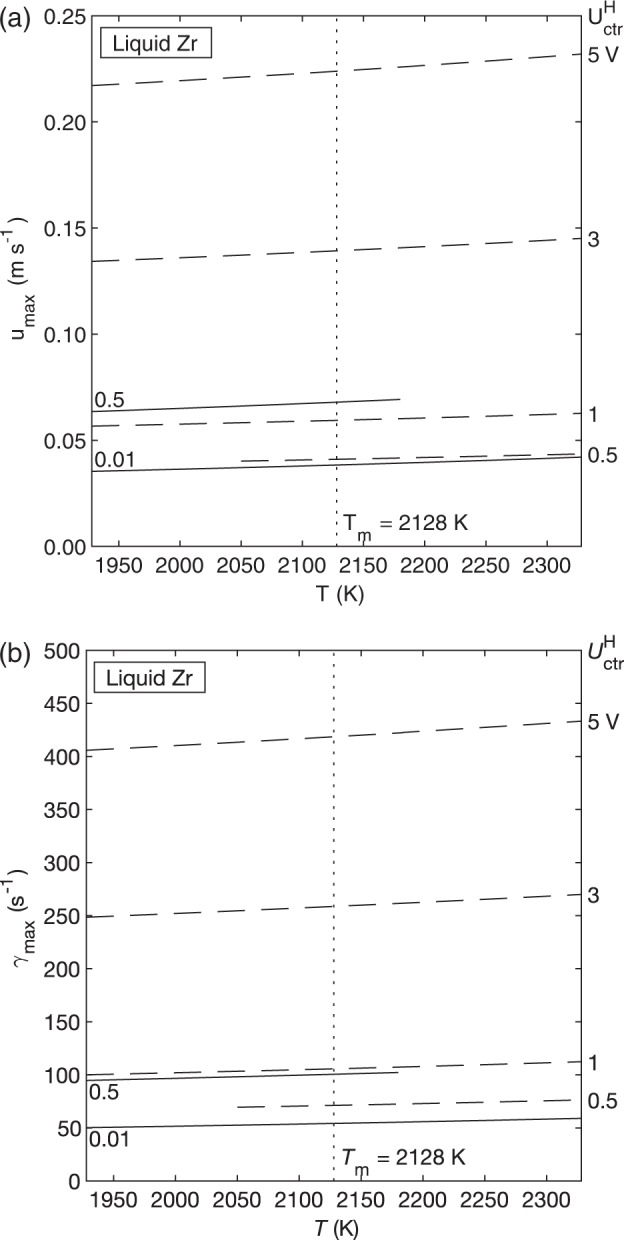
Convection in Zr. Maximum velocity (**a**) and maximum shear rate (**b**) vs. temperature as a function of heater control voltage. Solid lines are results from the laminar model (valid in laminar flow) and dashed lines are results from the turbulent model (valid in turbulent flow). Curves overlap in the transition region. Convection in the Zr sample increases as temperature and heater voltage increase.

In addition to the flow regime, we can also calculate *u*_max_ and $\dot \gamma _{{\mathrm{max}}}$ to predict the magnitude of convection in our samples when the heater is on (Fig. 6). Again, laminar flow is accessible in Zr if heater control voltage can be kept very low. We also observe that temperature has a minor effect on convection in Zr when compared to the effect of heater voltage.

While the surrogate model shows that laminar flow will result at certain combinations of heater voltage and temperature, we also must recognize that heater voltage and temperature are directly linked, meaning that not all combinations of these two parameters are possible in an experiment. For example, the surrogate model indicates that flow in Zr is laminar for isothermal hold at *T* = 2300 K and $U_{{\mathrm{ctr}}}^{\mathrm{H}}$ = 0.01 V, however, it is likely not possible to maintain the sample at 2300 K with such a small heater voltage. Thus, we employ the EML Experiment Simulator to inform our choice of heater voltage and temperature inputs to the surrogate model.

The EML Experiment Simulator can be used to calculate the heater voltage required to achieve a desired isothermal hold. These heater voltage and temperature pairs are then input to the surrogate model to calculate Reynolds number, *u*_max_, and $\dot \gamma _{{\mathrm{max}}}$ (Table 1). Results were calculated for isothermal holds in helium, argon, and vacuum environments. The main finding is that the required heater voltages for relevant isothermal-hold temperatures in Zr are too high to allow for the possibility of laminar flow. Even in vacuum, with 300 K undercooling, the necessary heater voltage is 3.76 V, and the predicted Re is 1018.1, well above the transition to turbulence at Re = 600. In this way, the EML Experiment Simulator can be used in concert with surrogate modeling to narrow down the range of allowable temperature and heater voltage and calculate the magnitude of convection under those conditions.

**Table 1:** EML Experiment Simulator results for Zr isothermal hold.

Hold temperature (K)		$U^{\rm{H}}_{\rm{ctr}}$ (V)	*u*_max_ (m s^−1^)	γ̇_max_ (s^−1^)	Reynolds number
*Helium*					
*T*_m_ + 200	2328	9.40	0.400	697.5	4165.6
*T*_m_ + 100	2228	8.70	0.372	663.6	3411.7
*T*_m_	2128	8.02	0.344	623.2	2816.7
*T*_m_ − 100	2028	7.36	0.315	578.2	2336.5
*T*_m_ − 200	1928	6.72	0.287	530.4	1942.5
*T*_m_ − 300	1828	6.10	0.259	481.2	1615.3
*Argon*					
*T*_m_ + 200	2328	7.25	0.325	594.7	3386.6
*T*_m_ + 100	2228	6.58	0.294	543.0	2691.7
*T*_m_	2128	5.93	0.263	489.3	2153.4
*T*_m_ − 100	2028	5.31	0.233	436.0	1730.3
*T*_m_ − 200	1928	4.72	0.205	384.1	1392.8
*T*_m_ − 300	1828	4.14	0.179	333.1	1115.7
*Vacuum*					
*T*_m_ + 200	2328	6.90	0.311	572.4	3243.3
*T*_m_ + 100	2228	6.23	0.279	518.5	2561.4
*T*_m_	2128	5.57	0.248	462.6	2031.2
*T*_m_ − 100	2028	4.94	0.218	407.5	1615.8
*T*_m_ − 200	1928	4.34	0.190	354.3	1285.6
*T*_m_ − 300	1828	3.76	0.163	303.3	1018.1

**Fig. 7:**
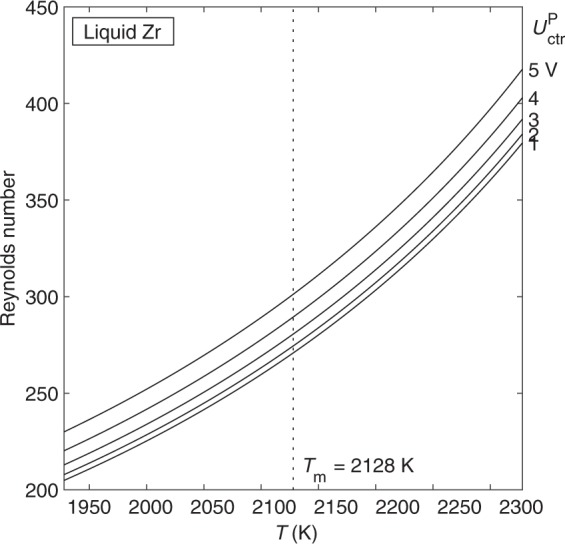
Reynolds number vs. temperature for flow in liquid Zr as a function of positioner control voltage, as predicted by the laminar model. The flow will be laminar for the full range of temperature and positioner voltage shown.

Although laminar flow is ultimately not accessible in Zr with the heater on for isothermal hold, we find that it predominates when the heater is off (Fig. 7). The flow in Zr is predicted to be laminar for all temperatures and positioner voltage levels shown. Free-cooling experiments on Zr are therefore expected to be straightforward to perform, as flow in the sample will be laminar through the entire cooling curve.

### Ti_39.5_Zr_39.5_Ni_21_

**Fig. 8:**
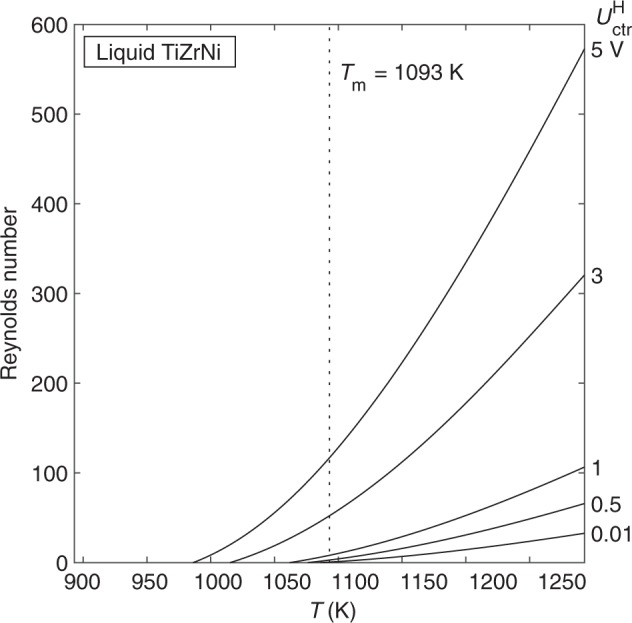
Reynolds number vs. temperature for flow in liquid TiZrNi as a function of heater control voltage, as predicted by the laminar model. The flow will be laminar for the full range of temperature and positioner voltage shown. Reynolds number drops to zero as the TiZrNi sample undercools due to a large increase in viscosity.

Finally, we examine the flow in Ti_39.5_Zr_39.5_Ni_21_. The Reynolds number is less than 600 for all experimental conditions shown, meaning that flow in TiZrNi will be laminar even when the heater control is on at low to moderate voltage (Fig. 8).

**Fig. 9:**
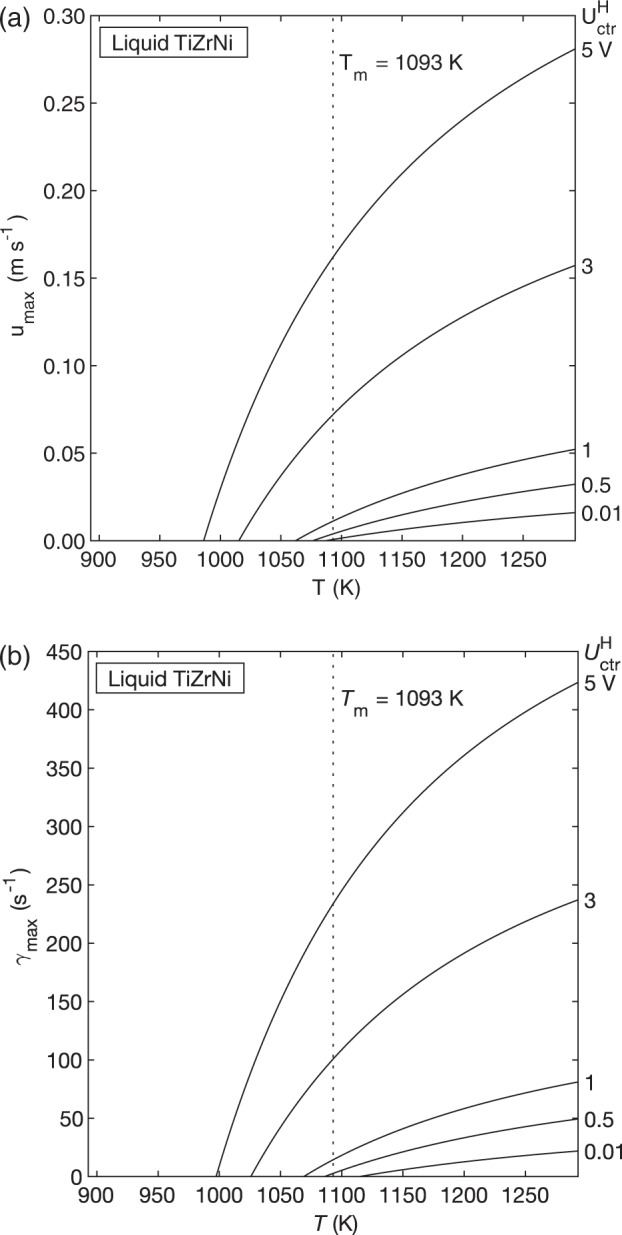
Convection in TiZrNi. Maximum velocity (**a**) and maximum shear rate (**b**) vs. temperature as a function of heater control voltage, as predicted by the laminar model. Convection in the TiZrNi sample increases as temperature and heater voltage increase. u_max_ and $\dot \gamma _{{\mathrm{max}}}$ drop to zero as the sample undercools due to a large increase in viscosity.

The surrogate model is once again used to calculate *u*_max_ and $\dot \gamma _{{\mathrm{max}}}$ as a function of temperature and heater voltage (Fig. 9). As expected, *u*_max_ and $\dot \gamma _{{\mathrm{max}}}$ increase with increasing temperature and heater voltage. However, we also see that *u*_max_ and $\dot \gamma _{{\mathrm{max}}}$ drop to zero as the sample undercools. This is due to a sharp increase in the viscosity of TiZrNi in the undercooled region. Therefore, the TiZrNi sample is predicted to become quiescent as temperature decreases and can even be quiescent above *T*_m_ if a very low heater voltage can be used.

Given that the flow in TiZrNi will be laminar under a wide range of conditions, investigators have more freedom to select experimental conditions that will produce a desired convection magnitude. The combined EML Experiment Simulator and surrogate model approach is used again for TiZrNi (Table 2). The isothermal-hold temperatures and associated heater control voltage lead to Reynolds numbers that are well within the laminar region. Thus, the gas environment, hold temperature, and heater voltage can be varied to set the desired convection magnitude for the experiment. Deeper undercoolings than those shown in the table may be achievable, but the TiZrNi sample will be quiescent under those temperature and heater voltage combinations.

**Table 2:** EML experiment simulator results for Ti_39.5_Zr_39.5_Ni_21_ isothermal hold.

Hold temperature (K)		$U^{\rm{H}}_{\rm{ctr}}$ (V)	*u*_max_ (m s^−1^)	γ̇_max_ (s^−1^)	Reynolds number
*Helium*					
*T*_m_ + 200	1293	4.02	0.220	329.8	448.2
*T*_m_ + 100	1193	3.53	0.154	228.9	206.6
*T*_m_ + 50	1143	3.29	0.116	170.6	118.4
*T*_m_	1093	3.06	0.074	104.2	53.7
*T*_m_ − 50	1043	2.83	0.025	24.4	11.8
*Argon*					
*T*_m_ + 200	1293	1.87	0.094	143.8	191.1
*T*_m_ + 100	1193	1.5	0.055	84.2	73.8
*T*_m_ + 50	1143	1.32	0.035	52.5	35.5
*T*_m_	1093	1.15	0.014	18.8	10.1
*Vacuum*					
*T*_m_ + 200	1293	1.31	0.066	102.4	134.7
*T*_m_ + 100	1193	0.94	0.034	53.0	46.3
*T*_m_ + 50	1143	0.76	0.019	28.6	19.7
*T*_m_	1093	0.59	0.005	4.5	3.5

## Discussion

To predict convection in an EML sample, we suggest a step-by-step procedure using the surrogate model as a planning tool. First, the temperature-dependent density, viscosity, and conductivity data for the material should be collected through literature review. Clearly, better property data will yield better results, however, the model can still provide insight when the data are uncertain. The maximum velocity and shear rate can then be calculated under heater or positioner control, starting with the laminar version of the model. The Reynolds number is then computed from the *u*_max_ result and the temperature-dependent density and viscosity. The predicted Reynolds number is itself useful information for planning experiments as thermophysical property measurements should ideally be made when flow in the droplet is laminar. If the Reynolds number indicates that flow is laminar, then the correct *u*_max_ and $\dot \gamma _{{\mathrm{max}}}$ have already been calculated. On the other hand, if the flow is turbulent, the model should be re-run with the turbulent model coefficients to obtain accurate values for *u*_max_ and $\dot \gamma _{{\mathrm{max}}}$ . In deciding between the laminar or turbulent model, we note that Re < 500 is merely a guide for laminar flow and researchers may adjust this criterion if more information is available.

Although the model produces *u*_max_ and $\dot \gamma _{{\mathrm{max}}}$ for any combination of temperature and heater voltage, it is important to note that only a subset of the temperature-voltage space is accessible. This is because the sample temperature directly depends on the heater voltage. Therefore, experiments parameters should be more fully constrained with the EML Experiment Simulator. In total, the investigator can estimate the heater voltage required for each thermal hold, and the velocity, shear rate, and Reynolds number to expect in the droplet at that point.

Ultimately, this convection information is directly applicable to two common types of EML experiments: free cooling and isothermal holds. In free cooling, the sample is melted and superheated before the heater control is turned off and the sample cools freely to the ambient. While cooling, the sample is under the influence of the position controller only, and convection is significantly reduced. Thus, for many materials, laminar flow may only be achievable in a free-cooling type experiment, as illustrated by our Zr results. The surrogate model describes how stirring in the sample changes with temperature, meaning that stirring magnitude during property measurement can now be known. Future studies may be able to correct for the influence of convection on their measurements, improving accuracy. For solidification studies, the model can also quantify the convection at the time of solidification, which is recognized as a key variable^6,17^.

In isothermal-hold experiments, the sample is melted and then held at constant temperature (above or below *T*_m_) with the heater control. Although this experiment type is essential for determining material properties at a given temperature, the strong influence of the heater causes greatly increased stirring. In many materials, the stirring will be strong enough to induce turbulence, which complicates analysis of measurement error and may invalidate the result. The surrogate model allows researchers to identify materials that will be viable candidates for isothermal-hold experiments, such as the TiZrNi composition shown here. By eliminating poor candidate materials in the planning stage, significant time and money can be saved.

The MHD model is steady-state, so researchers should be aware of the uncertainty in using the surrogate model to predict convection in a sample that is responding to changes in heater voltage and temperature. Assuming Fo ≅ 1, the time for internal thermal equilibrium is at most 2.0 s for the range of temperature in these experiments^11^. The error in sample temperature depends on the cooling rate, as Δ*T* = (*dT*/*dt*)Δ*t*. Thus, for a cooling rate in argon of 20 K s^−1^, Δ*T* = 40 K. In addition, the model assumes that the sample is experiencing limited oscillations and deformations, so it may not be appropriate for applications involving induced pulses for property measurement^12^.

We have modeled flow in Au, Zr, and TiZrNi in EML to predict convection conditions that affect measurement feasibility. Our results indicate that Au will be turbulent under most experimental conditions regardless of whether the heater is on or off, which presents a challenge to investigators. For instance, turbulent flow complicates the analysis of oscillation damping during viscosity measurement as turbulent eddies enhance momentum transfer thus reducing damping times^8^. We note that the model used in this study is specifically developed for ISS-EML and that model results do not apply to other EML facilities or competing measurement techniques. Nevertheless, researchers should be aware of this potential source of additional uncertainty.

Zr is predicted to be turbulent under accessible conditions with the heater on and laminar with the heater off. For isothermal-hold experiments, we therefore have the same reservations about Zr as we do for Au. Importantly, Zr will be laminar in heater-off, free-cooling experiments, so investigators are advised to restrict their study to that type, or at least be watchful for the onset of turbulence when the heater is on.

In contrast to Au and Zr, TiZrNi is an excellent candidate for EML experiments. Reynolds number for TiZrNi was less than 600 under all conditions modeled in this study. When not restricted by turbulence, the surrogate model is a powerful tool for tailoring experiment parameters to create a desirable amount of stirring. We can also compare convection for experiments done in different conditions. For example, for TiZrNi in a thermal hold at *T*_m_, $\dot \gamma _{{\mathrm{max}}}$ in helium is 5.5 times greater than $\dot \gamma _{{\mathrm{max}}}$ in argon and 23.1 times greater than $\dot \gamma _{{\mathrm{max}}}$ in vacuum (Table 2). In this way, differences in convection conditions can be quantified and used to examine discrepancies in experimental data.

**Fig. 10:**
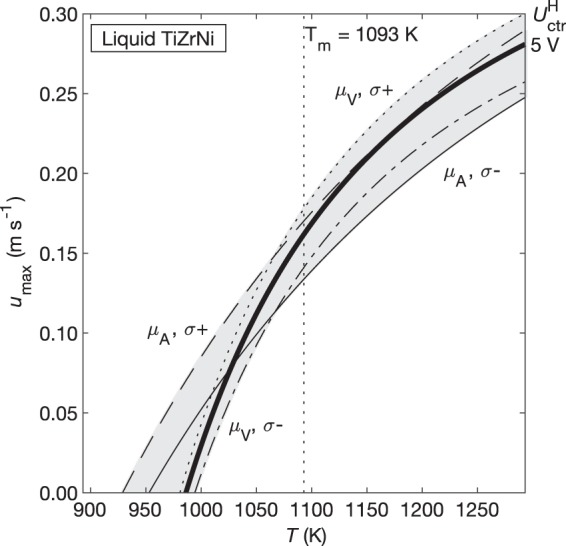
Sensitivity analysis of uncertainty in TiZrNi thermophysical properties. Bold solid line indicates best data available. Gray envelope marks maximum and minimum values of u_max_ based on the uncertainty in viscosity and electrical conductivity. Only the $U_{{\mathrm{ctr}}}^{\mathrm{H}}$ = 5 V line is shown to preserve clarity. Uncertainty envelope shrinks with decreasing heater voltage. Dotted: VFT fit for µ, σ + 20%. Dashed: Arrhenius fit for µ, σ + 20%. Dash-dot: VFT fit for µ, σ −20%. Solid thin: Arrhenius fit for µ, σ − 20%.

The surrogate model requires density, viscosity, and electrical conductivity data on the material of interest. Because EML experiments are often intended to measure these properties, it is reasonable to assume that some of this data will be unknown or uncertain during the experiment planning process. In this section we will demonstrate how this approach can be used even when the existing thermophysical property data is incomplete. The preceding TiZrNi results were generated using the best available data, as listed in Table 3^18–23^. However, other datasets exist for Ti_39.5_Zr_39.5_Ni_21_ for density, viscosity, and electrical conductivity. One area of uncertainty is in the viscosity at deep undercooling, which can be described by an Arrhenius fit instead of the VFT fit used here. Another uncertainty is in the electrical conductivity data, which lacked the appropriate calibration measurement during data collection. This calibration issue is estimated to introduce an error of ±10%, but we will use ±20% to take a more conservative approach. Multiple density datasets agree to within 1%, so the density error is not shown in the following analysis. The uncertainty in the viscosity and electrical conductivity data is used to make four alternate results which together represent the total uncertainty envelope of *u*_max_ (Fig. 10). The uncertainty varies with temperature, but, on average, is ±15% of the result produced by the best available data. This shows that a reasonable estimate of the flow conditions can be produced by the surrogate model even when there is significant uncertainty in the existing thermophysical property data.

**Table 3:** Thermophysical properties of Au, Zr and Ti_39.5_Zr_39.5_Ni_21_.

Material	*T*_m_ (K)	*ρ* (kg m^−3^)	*µ* (Pa s)	*σ*_el_ (S m^−1^)
Au	1337	19,300 (1.13 × 10^−8^ T^2^ + 6.16 × 10^−5^ T + 1.03)^−1 ^^18^	$\mu _0{\mathrm{exp}}(2.65T_m^{1.27}/(RT))$^19^	(2.10 × 10^−14^ T^2^ + 1.13 × 10^−10^ T + 1.47 × 10^−7^)^−1 ^^18^
Zr	2128	−0.27 (*T* − *T*_m_) + 6090^ 20^	−5.31 × 10^−6^(*T*−*T*_m_) + 4.83 × 10^−3 ^^21^	(8.74 × 10^−11^ T + 1.26 × 10^−6^)^−1 ^^18^
Ti_39._Zr_39.5_Ni_21_	1093	−0.188*T* + 6162^ 22^	*μ*_0_exp (*D*_VFT_*T*_0_/(*T* − *T*_0_))^23^^a^	(4.38 × 10^−13^ T^2^−1.41 × 10^−9^ T + 2.86 × 10^−6^)^−1^ [unpublished data]^b^

^a^Reference uses a Vogel-Fulcher-Tammann (VFT) fit and an Arrhenius fit. We use the VFT fit in our calculations as it better represents the glass-forming properties of TiZrNi. µ_0_ = 0.00225 Pa*s, D_VFT_ = 1.88, *T*_0_ = 686.25 K.

^b^Data from ISS EML Batch 2 resistivity measurement.

Given the need for accurate thermophysical properties and the growing list of materials to test, researchers conducting EML experiments have more candidate materials than time to test them. Determining which samples to run and how to conduct experiments efficiently is a critical way to accelerate the property measurement process. The modeling results presented here illustrate a method for doing exactly that. For Au, Zr, and Ti_39.5_Zr_39.5_Ni_21_ we show predicted convection conditions as a function of temperature and control voltage and discuss the implications for their viability in EML. Our main findings are summarized below:

1. Au will be turbulent under most operating conditions whether the heater is on or off. Laminar flow is only possible with the heater off, low positioner voltage, and significant undercooling. This presents significant challenges for accurate property measurement of Au in EML.
2. Zr will be turbulent with the heater on and laminar with the heater off. This precludes property measurement during thermal hold experiments, but free-cooling experiments should provide appropriate flow conditions for measurement. The convection magnitude through the entire cooling curve can be calculated.
3. Ti_39.5_Zr_39.5_Ni_21_ will be laminar under all operating conditions examined in this study, making it an excellent candidate for isothermal-hold and free-cooling EML experiments. The sample becomes quiescent as it undercools due to a large increase in viscosity. Sensitivity analysis for this sample shows that convection magnitude can be estimated even when there is significant uncertainty in the existing thermophysical property data.
4. Regardless of the viability of property measurement, solidification studies can benefit from the quantitative convection information provided by the model.

The surrogate model is easily applicable to other materials, without the need for computationally intensive simulation, enabling an efficient and informed approach to material selection and experiment planning for EML.

## Methods

### MHD surrogate model

The MHD simulation was performed for two different conditions for an ISS-EML sample. For the first condition, the heater was switched off and only the positioner voltage was controlled to simulate testing during free-cooling for dynamic property measurement or solidification undercooling experiments. For the second condition, the positioner control voltage was kept constant and the heater was controlled to simulate testing during a thermal hold or during slow cooling procedures. Both models include parametric input of positioning control voltage $U_{{\mathrm{ctr}}}^{\mathrm{P}}$, heater control voltage $U_{{\mathrm{ctr}}}^{\mathrm{H}}$, sample density *ρ*, viscosity *µ*, and electrical conductivity *σ*_el_. Droplet dimension has significant impact on shear rate and maximum convective velocity^24^ and is set to 6.5 mm in this study. The range of operating conditions for the generalized model is listed in Table 4.

**Table 4:** Sampling of parametric inputs of the MHD simulation.

Properties	Values
$U_{{\mathrm{ctr}}}^{\mathrm{P}}$: Positioning control voltage (V)	2.0–10.0
$U_{{\mathrm{ctr}}}^{\mathrm{H}}$: Heater control voltage (V)	0.01–6.00
*σ*_el_: Electrical conductivity (S m^−1^)	2.0 × 10^5^–6.0 × 10^6^
*ρ*: Density (kg m^−3^)	5000–10,000
*μ*: Viscosity (Pa s)	0.001–0.040

The max flow velocity and shear rate under various operating conditions for arbitrary droplet melts can be predicted using the surrogate model, developed by Xiao et al.^10,12^. In surrogate modeling, a polynomial function is created by mapping input parameters to model results.

### Case 1: Positioner only

The following equation was developed to calculate both *u*_max_ and $\dot \gamma _{{\mathrm{max}}}$ in laminar flow by keeping the heater off and varying the positioning control voltage^10^:

1 $$u_{\mathrm{max}}\,{\mathrm{or}}\,\dot \gamma _{\mathrm{max}} = \exp \left( {\mathop {\sum }\limits_{i,j,k,s} p_{ijks}.(U_{{ctr}}^P)^i.\left( {\frac{1}{{\sqrt {\sigma _{\mathrm{el}}} }}} \right)^j.\rho ^k.\left( {\frac{1}{{\ln \left( \mu \right)}}} \right)^s} \right)$$

where the coefficients *p*_*ijks*_ were derived using least-squared fitting and feature selection from raw data. The *p* coefficients for Eq. (1) are listed in Supplementary Table 1. Note that *i* tracks the contribution by the positioning control voltage, *j* tracks the electrical conductivity, *k* tracks the density, and *s* tracks the viscosity.

### Case 2: Heater on

For the common operating range, the heating field is much stronger than the positioner field, so the effect of positioner voltage can be neglected when the heater is on. The following equation was developed to calculate both velocity and shear rate by varying heater control voltage with constant positioner control voltage^12^,

2 $$u_{\mathrm{max}}\,{\mathrm{or}}\,\dot \gamma _{\mathrm{max}} = \mathop {\sum }\limits_{i,j,k,s} q_{ijks}.\left( U_{\mathrm{ctr}}^{\mathrm{H}} \right)^i.\rho ^j.\left( {\ln \left( \mu \right)} \right)^k.\left( {\ln \left( {\sigma _{\mathrm{el}}} \right)} \right)^s$$

The q coefficients for Eq. (2) are listed in Supplementary Table 2. Note the variable notation is different for the heating field as compared to the positioning field: *i* tracks the contribution by the heating control voltage, *j* tracks the density, *k* tracks the viscosity, and *s* tracks the conductivity.

The Reynolds number can be calculated from the density, viscosity, and maximum velocity at a particular temperature:

3 $${\mathrm{Re}} = \frac{{\rho u_{\mathrm{max}}D}}{\mu }$$

where *D* is the droplet diameter. Re is used as a criterion to identify the flow conditions, where the flow is laminar for Re < 500, transitions to turbulent for 500 < Re < 600, and is fully turbulent for Re > 600^12^. Equations (1)–(3) can directly be used for predicting the outcome of future experiments in ISS-EML facility without running the entire flow simulation.

### Example calculation

Equation (2) can be used to predict *u*_max_ as a function of *ρ*, *µ*, and *σ*_el_ for $U_{{\mathrm{ctr}}}^{\mathrm{H}}$ = 5.0 V. The values of *ρ*, *µ*, and *σ*_el_ as a function of temperature should be obtained from the literature.

In Eq. (2), the first term of the summation is obtained by setting *i* = 0, *j* = 0, *k* = 0 and *s* = 0,

1st term: $$u_{\mathrm{max},1} = q_{0000}(U_{\mathrm{ctr}}^{\mathrm{H}})^0\rho ^0\left( {\ln \mu } \right)^0(\ln \sigma _{\mathrm{el}})^0 = q_{0000} = 2.705 \times 10^1$$

The second term of the summation is obtained by setting *i* = 0, *j* = 0, *k* = 0 and *s* = 1,

2nd term: $$u_{\mathrm{max},2} = q_{0001}(U_{\mathrm{ctr}}^{\mathrm{H}})^0\rho ^0\left( {\ln \mu } \right)^0(\ln \sigma _{\mathrm{el}})^1 = - 2.375 \times 10^{ - 2}(\ln \sigma _{\mathrm{el}})$$

Similarly, all 17 terms can be calculated using the values listed in Supplementary Table 2:

$$\begin{array}{l} u_{\mathrm{max}}=q_{0000} + q_{0001}(\ln\sigma _{\rm{el}}) + q_{0010}\left( \ln \mu \right) + q_{0011}\left(\ln \mu \right)(\ln \sigma _{\mathrm{el}}) + q_{0012}\left( \ln\mu \right)\left(\ln \sigma _{\mathrm{el}} \right)^{2}\\+\; q_{0021}\left(\ln \mu \right)^{2}\left(\ln \sigma_{\mathrm{el}}\right) + q_{1000}U_{\mathrm{ctr}}^{\mathrm{H}}+q_{1001}U_{{\mathrm{ctr}}}^{\mathrm{H}}\left(\ln \sigma_{\mathrm{el}}\right) +q_{1002}U_{\mathrm{ctr}}^{\mathrm{H}}\left(\ln \sigma _{\mathrm{el}}\right)^2\\+\; q_{1010}U_{\mathrm{ctr}}^{\mathrm{H}}\left(\ln \mu \right) +q_{1011}U_{\mathrm{ctr}}^{\mathrm{H}}\left(\ln \mu \right)\left(\ln \sigma _{\mathrm{el}}\right) +q_{1020}U_{\mathrm{ctr}}^{\mathrm{H}}\left(\ln \mu \right)^{2} +q_{1100}U_{\mathrm{ctr}}^{\mathrm{H}}\rho\\+\;q_{1101}U_{\mathrm{ctr}}^{\mathrm{H}}\rho \left( \ln \sigma_{\mathrm{el}} \right) + q_{1110}U_{\mathrm{ctr}}^{\mathrm{H}}\rho\left(\ln \mu \right) + q_{1200}U_{\mathrm{ctr}}^{\mathrm{H}}\rho^{2} +q_{2000}(U_{\mathrm{ctr}}^{{\mathrm{H}}})^{2}\\+\;q_{2001}(U_{\mathrm{ctr}}^{\mathrm{H}})^{2}\left(\ln \sigma _{\rm{el}}\right) +q_{2010}(U_{\mathrm{ctr}}^{\mathrm{H}})^{2}\left(\ln \mu\right)+ q_{2100}(U_{\mathrm{ctr}}^{\mathrm{H}})^{2}\rho +q_{3000}(U_{\mathrm{ctr}}^{\mathrm{H}})^{3}\end{array}$$

For a 6.5 mm zirconium (*T*_m_ = 2128 K) sample at *T* = 2200 K the density is 6070.6 kg m^−3^, viscosity is 0.0044 Pa s, and electrical conductivity is 68,957 S m^−1^. For this particular sample, the maximum convective velocity is:

$$\begin{array}{*{20}{l}} { u_{\mathrm{max}}} \hfill & = \hfill & {2.705 \times 10^1 - 2.375 \times 10^{ - 2}(\ln \sigma _{\mathrm{el}}) + 1.481 \times 10^{ - 1}\left( {\ln \mu } \right) - 1.758} \hfill \\ {} \hfill & {} \hfill & { \times 10^{ - 2}(\ln \sigma _{\mathrm{el}}) + 3.930 \times 10^{ - 4}\left( {\ln \mu } \right)\left( {\ln \sigma _{\mathrm{el}}} \right)^2 - 1.420} \hfill \\ {} \hfill & {} \hfill & { \times 10^{ - 5}(\ln \sigma _{\mathrm{el}}) - 9.354 \times 10^{ - 1}\left( {\ln \mu } \right)^2\left( {\ln \sigma _{{\mathrm{el}}}} \right) + 1.100 \times 10^{ - 1}(U_{\mathrm{ctr}}^{\mathrm{H}}) - 3.587} \hfill \\ {} \hfill & {} \hfill & { \times 10^{ - 3}\left( {U_{\mathrm{ctr}}^{\mathrm{H}}} \right)\left( {\ln \sigma _{\mathrm{el}}} \right) - 3.068 \times 10^{ - 3}\left( {U_{\mathrm{ctr}}^{\mathrm{H}}} \right)\left( {\ln \sigma _{el}} \right)^2 - 1.638} \hfill \\ {} \hfill & {} \hfill & { \times 10^{ - 3}\left( {U_{\mathrm{ctr}}^{\mathrm{H}}} \right)\left( {\ln \mu } \right) - 2.927 \times 10^{ - 3}\left( {U_{\mathrm{ctr}}^{\mathrm{H}}} \right)\left( {\ln \mu } \right)\left( {\ln \sigma _{\mathrm{el}}} \right) + 9.942} \hfill \\ {} \hfill & {} \hfill & { \times 10^{ - 6}\left( {U_{\mathrm{ctr}}^\mathrm{H}} \right)\left( {\ln \mu } \right)^2 - 8.575 \times 10^{ - 7}\left( {U_{\mathrm{ctr}}^\mathrm{H}} \right)\rho + 1.135 \times 10^{ - 6}\left( {U_{\mathrm{ctr}}^\mathrm{H}} \right)\rho \left( {\ln \sigma _{\mathrm{el}}} \right)} \hfill \\ {} \hfill & {} \hfill & { + 2.931 \times 10^{10}\left( {U_{\mathrm{ctr}}^{\mathrm{H}}} \right)\rho \left( {\ln \mu } \right) + 3.861 \times 10^{ - 3}(U_{\mathrm{ctr}}^{\mathrm{H}})\rho ^2 + 7.098 \times 10^4\left( {U_{\mathrm{ctr}}^{{\mathrm{H}}}} \right)^2} \hfill \\ {} \hfill & {} \hfill & { + 1.269 \times 10^{ - 3}\left( {U_{\mathrm{ctr}}^{{\mathrm{H}}}} \right)^2\left( {\ln \sigma _{\mathrm{el}}} \right) - 7.639 \times 10^{ - 8}\left( {U_{\mathrm{ctr}}^{{\mathrm{H}}}} \right)^2\left( {\ln \mu } \right) - 6.038} \hfill \\ {} \hfill & {} \hfill & { \times 10^{ - 4}\left( {U_{\mathrm{ctr}}^{{\mathrm{H}}}} \right)^2\rho } \hfill \\ {} \hfill & = \hfill & {0.2266\,{\mathrm{m}}\,{\mathrm{s}}^{ - 1}} \hfill \end{array}$$

The Reynolds number for this case is 3618 which is in the turbulent regime. Calculating convection as a function of temperature requires data on density, viscosity, and electrical conductivity as a function of temperature. The available literature data is listed in Table 3. We employ the surrogate model for the temperature range *T*_m_ ± 200, but acknowledge that much of the literature data is collected over a more limited temperature range. Analysis of the uncertainty in the surrogate model when the material property data is not fully known is included in the discussion.

We use a MATLAB script to perform the surrogate model calculations. Additional tools include the MSL/EML Experiment Simulator developed by DLR and S.E.A., which predicts temperature-time profiles of EML samples using RF parameters and coupling coefficients that describe the interaction of the sample with the RF field^25^. Parameter sets for Zr and TiZrNi for the Experiment Simulator were provided by DLR.

### Reporting summary

Further information on research design is available in the Nature Research Reporting Summary linked to this article.

## Supplementary information

Supplementary Information

Reporting Summary

## Data Availability

The data that support the findings of this study are available from the corresponding author upon reasonable request.
