# Supplementary material for: MHD surrogate model for convection in electromagnetically levitated molten metal droplets processed using the ISS-EML facility
Source: NPJ Microgravity. 2020 Mar 16;6:9. doi: 10.1038/s41526-020-0099-7 (PMC7076000; doi:10.1038/s41526-020-0099-7)
Supplement: Supplementary file 1 — Supplementary Information [file 41526_2020_99_MOESM1_ESM.pdf]

Supplementary Table 1 Model coefficients for positioner-controlled flow *[10]*

p_{ijks}	$u_{\max}$ (m s^{-1})	$\dot{\gamma}_{\max}$ (s^{-1})
p_{0000}	-2.346	4.262
p_{0001}	-2.372×10^{1}	-2.412×10^{1}
p_{0002}	-1.702×10^{2}	-1.746×10^{2}
p_{0003}	-2.436×10^{2}	-1.746×10^{2}
p_{0010}	-1.806×10^{-4}	-1.644×10^{-4}
p_{0011}	-9.965×10^{-4}	-7.778×10^{-4}
p_{0012}	-1.380×10^{-3}	-7.124×10^{-4}
p_{0100}	-1.025×10^{3}	1.259×10^{3}
p_{0101}	-1.149×10^{2}	-6.587×10^{3}
p_{0200}	4.613×10^{5}	-1.007×10^{6}
p_{0201}	7.176×10^{5}	-1.420×10^{6}
p_{0300}	-8.444×10^{7}	1.864×10^{8}
p_{1000}	-9.751×10^{-3}	-4.866×10^{-3}
p_{1001}	-7.632×10^{-2}	-1.070×10^{-1}
p_{1100}	-1.725×10^{1}	2.389
p_{1101}	2.702×10^{1}	4.944×10^{1}
p_{1200}	4.975×10^{3}	2.067×10^{2}
R^2	0.9960	0.9930

Supplementary Table 2 Model coefficients for heater-controlled flow *[12]*

	Laminar model		Turbulent model	
q_{ijks}	$u_{\max}$ (m s^{-1})	$\dot{\gamma}_{\max}$ (s^{-1})	$u_{\max}$ (m s^{-1})	$\dot{\gamma}_{\max}$ (s^{-1})
q_{0000}	2.705×10^{-1}	-2.412×10^{1}	1.025×10^{-1}	2.213×10^{2}
q_{0001}	-2.375×10^{-2}	-3.601×10^{1}	-9.377×10^{-3}	-1.796×10^{1}
q_{0010}	1.481×10^{-1}	1.152×10^{2}	9.369×10^{-2}	1.4325×10^{2}
q_{0011}	-1.758×10^{-2}	-1.196×10^{1}	-1.221×10^{-2}	-1.662×10^{1}
q_{0012}	3.930×10^{-4}	3.746×10^{-2}	3.322×10^{-4}	4.002×10^{-1}
q_{0021}	-1.402×10^{-5}	-1.304×10^{-1}	-2.197×10^{-5}	-4.452×10^{-2}
q_{1000}	-9.354×10^{-1}	-1.634×10^{3}	-4.259×10^{-1}	-7.906×10^{2}
q_{1001}	1.100×10^{-1}	1.881×10^{2}	5.454×10^{-2}	8.993×10^{1}
q_{1002}	-3.587×10^{-3}	-5.873	-1.796×10^{-3}	-2.927
q_{1010}	-3.068×10^{-2}	-8.963×10^{1}	-1.167×10^{-3}	-1.992×10^{1}
q_{1011}	-1.638×10^{-3}	1.088×10^{-2}	1.233×10^{-3}	-2.826
q_{1020}	-2.927×10^{-3}	-5.758	-7.224×10^{-4}	-3.369
q_{1100}	9.942×10^{-6}	1.260×10^{-2}	4.693×10^{-6}	-1.031×10^{-2}
q_{1101}	-8.575×10^{-7}	-1.170×10^{-3}	-4.767×10^{-7}	-9.095×10^{-4}
q_{1110}	1.135×10^{-6}	1.635×10^{-3}	5.908×10^{-7}	1.245×10^{-3}
q_{1200}	2.931×10^{-10}	4.580×10^{-7}	1.775×10^{-10}	3.035×10^{-7}
q_{2000}	3.861×10^{-3}	6.457	-9.398×10^{-4}	1.933
q_{2001}	7.098×10^{-4}	7.534×10^{-1}	3.206×10^{-4}	6.064×10^{-1}
q_{2010}	1.269×10^{-3}	1.993	2.329×10^{-4}	9.055×10^{-1}
q_{2100}	-7.639×10^{-8}	-9.062×10^{-5}	-1.077×10^{-4}	3.413×10^{-6}
q_{3000}	-6.038×10^{-4}	4.668×10^{-1}	-1.623×10^{-4}	-4.845×10^{-1}
R^2	0.9963	0.9939	0.9982	0.9957
